# Lived Experience of Hereditary Chronic Pancreatitis – A Qualitative Interview Study

**DOI:** 10.1177/17423953211039774

**Published:** 2021-09-24

**Authors:** Regina Müller, Ali A Aghdassi, Judith Kruse, Markus M Lerch, Christoph Rach, Peter Simon, Sabine Salloch

**Affiliations:** 1 Institute of Ethics and History of Medicine, University of Tuebingen, Tübingen, Germany; 2 Institute of Ethics and History of Medicine, 60634 University Medicine Greifswald, Greifswald, Germany; 3 Department of Medicine A, 221223 University Medicine Greifswald, Greifswald, Germany; 4 Department of Psychiatry, Psychotherapy and Psychosomatics, 84491 Agaplesion Markus Hospital, Frankfurt am Main, Germany; 5 Institute of Ethics, 88782 History and Philosophy of Medicine, Hannover Medical School, Hannover, Germany

**Keywords:** Chronic illness, hereditary chronic pancreatitis, biographical disruption, ‘shifting perspectives model’, bioethics

## Abstract

**Objectives:**

Hereditary chronic pancreatitis is a rare condition characterized by intermittent acute episodes of pancreatitis and long-term impairment of pancreatic functions. However, the subjective perspective of individuals affected by hereditary chronic pancreatitis has been little studied. This qualitative study investigates the experience of hereditary chronic pancreatitis patients and their relatives because the awareness of the needs of those affected is an essential component of a patient-centered management of chronic conditions.

**Methods:**

Semi-structured qualitative interviews were conducted with hereditary chronic pancreatitis patients and their relatives. Data were analysed using qualitative content analysis. The concepts of ‘biographical contingency,’ ‘biographical disruption’ and the ‘shifting perspectives model’ served as theoretical frameworks.

**Results:**

A total of 24 participants (17 patients, 7 relatives) were interviewed individually. Four main themes were identified: (1) The unpredictable clinical course of hereditary chronic pancreatitis; (2) hereditary chronic pancreatitis as a devastating experience; (3) hereditary chronic pancreatitis as part of a normal life; and (4) being reduced to hereditary chronic pancreatitis.

**Discussion:**

The ‘shifting perspectives model’ of chronic illness covers the four dimensions adequately and can serve as a theoretical model to explain hereditary chronic pancreatitis patients’ experience. A better understanding of the patients and their families’ experience and the shifting character of hereditary chronic pancreatitis can help healthcare professionals to tailor the care to meet the needs of those affected.

## Introduction

As a basic prerequisite for effective chronic illness care, healthcare systems have to meet the needs of those who are affected.^1^ Frameworks for managing and improving chronic care processes, such as the Chronic Care Model (CCM) and its adaptation for international contexts, the Innovative Care for Chronic Conditions framework, have recommended care consistent with the patients’ preferences for more than two decades.^1,2^ According to the CCM, effective chronic illness care is, among others, based on the individualization of care according to patients’ needs and values.^1^ The implementation of the CCM can improve medical outcomes and enhance the health-related quality of life of patients with chronic illness, yet, there are some limitations of the CCM and knowledge gaps regarding the benefits and barriers during CCM implementation in different healthcare settings.^3^ Although the CCM has been criticized in different aspects, for example, its lack of attention to chronic multimorbidity^4^ and paediatric populations,^5^ and consequently expanded, for example in the Patient-Centered Medical Home Model,^6,7^ ‘the model still holds.’^8^

Its core components, emphasizing the individual needs and preferences of those affected and their self-management support, are still relevant subjects of current research on chronic conditions, for example, the barriers and facilitators to self-management in chronic illness^9^ or the potential improvements for patients through self-management support.^10^ The subjective perceptions of patients with chronic illness have become a relevant part of this research focusing, for example, on the quality of chronic illness care,^11^ the factors affecting self-management^9^ and the support of self-management.^12^ However, although the perspectives of patients and their needs have received increasing attention in both chronic illness care and research, many rare chronic conditions, such as hereditary chronic pancreatitis (HCP) and the specific needs associated, are still underexposed in research.

The current paper presents findings on the subjective experience of patients with HCP and their relatives as part of a larger research project on hereditary disorders of the pancreas and liver [http://www.medizin.uni-greifswald.de/peppp/index.php?id=522&L=1]. The study design has an explorative qualitative character because HCP patients and their relatives have received little systematic empirical scrutiny so far. The aim is to acquire a firsthand understanding of those living with HCP. The main research question is, therefore, how do the individuals affected (patients, partners and family members) experience HCP. The concepts of ‘biographical contingency’ and ‘biographical disruption’ and the ‘shifting perspectives model’ serve as theoretical frameworks.

## Hereditary chronic pancreatitis

Hereditary chronic pancreatitis (HCP) is a chronically progressive, rare variant of early-onset pancreatitis. Recurrent acute episodes of pancreatitis are accompanied by a persistent impairment of the exocrine and endocrine pancreatic function^13^ due to the loss of parenchymal tissue and the formation of fibrosis.^14^ The clinical symptoms can include abdominal pain, nausea and vomiting. Long-term complications are maldigestion and weight loss due to exocrine insufficiency, pancreoprive diabetes, that results from an impairment of endocrine function, and an increased risk of pancreatic cancer.^15,16^ Other common complications are pseudocyst formation,^17^ bile and pancreatic duct,^18^ as well as duodenal obstruction.^19^ Since there is no curative treatment for HCP currently, the therapy covers pain management, therapy for endocrine and exocrine insufficiency, and endoscopic or surgical treatment for bile or pancreatic duct stenosis or for the drainage of pancreatic pseudocysts.^19,20^ Diagnosis, prognosis and treatment are challenging, as the course of the disease ranges from asymptomatic to very severe forms.^21^

The variations in the clinical course of chronic (and acute) pancreatitis and their adverse impact on health-related quality of life, daily activities and social life have been investigated in a few qualitative studies.^22–24^ A recent phenomenological study, describing the patients’ perceptions of recovering from an acute pancreatic attack, emphasized the physical and emotional burdens, such as uncertainty and anxiety, in the context of an acute attack.^23^ Similar to acute attacks, the chronic form of pancreatitis is associated with psychological burdens for the patients affected.^25^ A qualitative study with chronic pancreatitis (CP) patients highlighted the permanent experience of suffering and disruption at the physiological and psychological levels.^22^ However, the uncertainties and worries surrounding the acute attacks affect not only the patients but also their relatives.^24^ Family members additionally describe the experience of seeing relatives affected by the hereditary form of pancreatitis as a disturbing experience.^26^

Although there is a considerable amount of qualitative research on acute^23^ and chronic pancreatitis,^22^ there has been far less qualitative research on patients’ experience with the hereditary variant of the disease. The concurrence of the dimensions *rare*, *hereditary* and *chronic* may lead to specific challenges for patients and their families, so that the existing research on acute and chronic pancreatitis and, accordingly, the therapy options and support available may not be directly transferable to HCP. Instead, the existing research needs to be expanded to give healthcare professionals a comprehensive picture of what needs to be done when they care for both patients with HCP and their relatives.

## Theoretical framework

The subjective experience of living with a chronic condition has received increasing research interest both in medicine and the sociology of health and illness since the 1980s.^27–35^ Ongoing debates on chronic illness focus on individual coping strategies,^35^ self-management,^36,37^ the consequences of a chronic illness for the identity of patients, especially of young patients,^38–40^ and the correlations to employment,^41^ family^42,43^ and social life.^44^

The concept of biographical disruption, according to Bury,^45^ often serves as a theoretical background for research on the subjective experience of chronic conditions. Bury conceptualizes chronic illness as a particular type of disruptive experience and argues that the onset of a chronic illness represents a biographical disruption, marking a life before and after illness.^45^ The concept of biographical disruption has been paradigmatic in the field of chronic illness studies for a few decades. The more recent literature, however, highlights its limitations and the need for more differentiated concepts, such as biographical reinforcement,^46^ biographical flow,^47^ recurrent biographical disruption^48^ or biographical contingency.^49^ The latter approach, for example, conceptualizes chronic illness as an ‘only sometimes problem’^49^ and describes living with a chronic illness to a large extent as normal and, simultaneously, attributes a disruptive potential to the illness.^49^

Although the research has become increasingly differentiated, many approaches have in common that they understand chronic conditions as predictable linear paths.^50^ However, the idea that a person with a chronic illness follows a trajectory is, in Paterson's opinion, misguiding and incomplete.^50^ Her ‘shifting perspectives model’ of chronic illness describes living with a chronic condition as an ongoing, continually changing process in which either elements of illness or wellness can be in the foreground.^50^ The perspective of the patient can shift from illness (i.e. illness dominates the daily life) to wellness (i.e. illness is largely unnoticed) and *vice versa*, for example, because the subjective illness experience or the social context changes.^50^ Due to the variation in the clinical course of HCP known from the literature, Paterson's account seems to be a suitable lens for the current study because of the possibility of variation and individualization of the illness experience.

## Methods

### Study design

The lack of research on the subjective experience of HCP in the literature influenced the development of the study aim and research question. Due to the gap, the aim of the present study is to acquire a firsthand understanding of those living with HCP. The main research question is, therefore, how do the individuals affected (patients, partners and family members) experience HCP? An exploratory qualitative design was chosen to clarify the relatively unknown experience of living with HCP.^51^ Qualitative semi-structured interviews were used because they allow one to elicit data grounded in the participants’ experience, while they retain some relation to the theories identified in the literature, namely, the concept of biographical disruption and the shifting perspectives model of chronic illness.

The development of the interview questions was carried out in a stepwise process. In the first step, based on the existing literature and the research team's experience, brainstorming was conducted to collect possible questions. In addition to the main research question of how those affected experience HCP, the theories identified in the literature led to further questions. The concept of biographical disruption, for example, which focuses on the onset of a chronic illness, raised questions about the diagnosis of HCP; the shifting perspectives model of chronic illness led to questions on the changes between ‘normal’ and ‘acute’ illness phases. In the second step, all questions collected were checked for their suitability, e.g. whether the questions were relevant to the objectives of the study. In the last step, the relevant questions were sorted and grouped into themes, e.g. in ‘changes of illness phases.’ The resulting interview guide starts with theoretically driven open-ended questions about the diagnosis of HCP, through questions about living with HCP to those about the changing illness phases, and ends with a more narrative question about the meaning of living with HCP for the person affected (Box 1).

Box 1. Interview questions (selection/version for patients).

**How did you realize that you have this disease?**

**The diagnosis is often a long process. Would you tell me something about it?**

 How did you realize you were ill?

 How/when did you hear that you have pancreatitis?

 Has something changed since the diagnosis?

 What happened after diagnosis?

**What is it like to live with the disease?**

 Changes between ‘normal’ and ‘acute’ illness phases?

**How are you doing with the disease right now?**

**Do you have any restrictions in your daily life?**

 Does the disease affect your education/job?

 Does the disease affect your family life?

**Would you complete the following sentence for me: Living with chronic pancreatitis means for me …**

Two slightly modified versions of the interview guide, one for patients and one for relatives, were developed. One interview with a patient and one with a relative as face-to-face pilots were conducted by RM, a female PhD student. These two interviews were included in the final analysis as the pilot test resulted only in minor modifications to the interview guides.

### Study participants

Both patients and their relatives were invited to participate in the current study since the family context has been proven to be a major factor in the context of chronic conditions.^24,26,42,43^ A patient organization for patients with HCP and their families in Germany (Deutsche Pankreashilfe e.V.) was involved to gain access to potential study participants. This organization has had a longstanding close relationship with two of the researchers (MML and PS). The chairperson of the organization forwarded an open invitation to participate in the interview study to the members by email and verbally at events arranged by the organization. Individuals who responded to these calls received written information about the context and objectives of the study by email and post. RM contacted those interested by telephone to clarify any remaining questions. Snowballing sampling was additionally used to locate further study participants, for example, individuals who are not members of the patient organization: Those contacted through the patient organization were asked whether they could forward the open invitation to others who could be interested in becoming study participants.

The sample was restricted to patients who self-identified as HCP patients, i.e. patients who had a personal history of pancreatitis and/or had been tested for the hereditary form (PRSS1 mutations) and/or already had HCP in their family (≥2 individuals with pancreatitis in ≥2 generations). Although HCP could not be verified in every patient by previous genetic test results, it was assumed because of the personal history of pancreatitis, the occurrence of HCP in the family and the absence of other explanatory etiologies (e.g. alcohol). Inclusion criteria regarding unaffected family members restricted the sample to the parents, children, siblings, aunts, uncles, spouses and life partners of HCP patients. The inclusion criterion, at least 18 years of age, applied to all participants. Variations in age, gender, educational level, marital status and the course of the disease were aimed for in the sampling.

### Data collection and analysis

The individual face-to-face interviews were conducted by RM (trained in empirical bioethics and qualitative research) at the participant's home. If a personal visit was difficult for the interview participant to arrange, telephone interviews were offered as a backup option. The same interview guide was used in the telephone interviews as in the face-to-face interviews, but the participants were contacted by telephone prior to the actual telephone interview to build trust and rapport and enable a free-flowing conversation. In order to gain the participant's full attention during the telephone interview, instructions were given in advance to provide enough time and a quiet room without potential disturbances.

All interviews (both the face-to-face and the telephone interviews) were fully audio-recorded, transcribed verbatim and pseudonymized. In addition to the audio recording, the interviewer made field notes during and after all interviews.

The interview transcripts were analysed using content-analytical procedures. The methodology selected for the data analysis was qualitative content analysis according to Mayring.^52^ Qualitative content analysis is a systematic data analysis technique. It was selected as the analytic method because it is independent of theoretical perspectives, very flexible and provides a systematic way of reducing and synthesizing a wide range of data.^53^ Its central idea is to assign categories to text passages through a qualitative-interpretative act.^52^ The analysis follows a systematic procedure and strict content-analytical rules combining deductive and inductive category development.^52^

Correspondingly, the transcripts were worked through with a previously developed, deductively formulated category system derived from theory. RM and SS categorized the interview text into clusters of conceptual categories with the aid of the deductively formulated category system and the software program MAXQDA12. Additionally, new categories were formulated out of the text. A coding scheme was created using the deductive and inductive category development and deliberated in recurring team meetings (for examples of the themes and (sub-)categories, see Table 1). Finally, the coding scheme was applied to all transcripts and the results were further interpreted regarding the categories generated. The team discussions and the different professional backgrounds of the researchers (medicine, philosophy and ethics) are intended to mitigate the rater influence.

**Table 1.** Themes and categories with examples.

Themes	Categories	Sub-categories	Representative quotes
Unpredictable clinical course of HCP	HCP as an ongoing but unstable condition	Episodic occurrence; disappearance; comparison with a cycle	*Yes, it does restrict me, but not as much as another illness that I would have all the time. Because in my case it only occurs in episodes and then it usually goes away again.(Interview 5)*
Unpredictable clinical course of HCP	Unpredictability; not knowing; fear of attacks; helplessness	Unpredictable clinical course; Russian roulette; disease not known; always expecting an attack; reason for the attack unknown; at the mercy of the disease	*Especially in the beginning, the first few years, it was unpredictable and because I didn’t know what I had, it was like a game of roulette or Russian roulette for me, where I always had to expect that I would be lying down the next day and that I wouldn’t know why and was at the mercy of it. (Interview 11)*
HCP as a devastating experience	Restrictions	Restrictions in general; effects in many areas; not being able to do things as wanted	*Well, for me, it means restrictions in many areas, you can’t do the things the way you want but, on the other hand, it's also a disease that you can definitely live with. (Interview 15)*

The present study is reported according to the COREQ checklist for qualitative research (Supplement 1) and was conducted in accordance with the Declaration of Helsinki. Written informed consent was obtained from all study participants and they were informed that study participation was voluntary. Other research ethics requirements, such as data protection, were followed diligently. The institutional Ethics Committee of the University Medicine Greifswald approved the study (ref. BB 074/17).

## Results

Twenty-six participants were enrolled in the interview study between July 2017 and December 2019. Two participants declined to be interviewed for personal reasons, resulting in a total of 24 individual interviews. Of these 24 interviews, 17 were with patients and 7 with relatives. Twenty-two participants were interviewed in their own homes; two interviews were conducted by telephone. The interviews lasted an average of 44 minutes (median: 43 minutes), ranging from 16 to 91 minutes.

Different stages of HCP were covered in the study. The patients had had a clinically overt condition since their birth, childhood or adulthood and one patient was in an acute phase of the condition during the interview process. In order to cope with complex familial relationships during the interview study, participants were asked to assign a role to themselves, which resulted in the three categories: Patient, partner and parent. Most participants were married, well-educated and more than 30 years old. Most of the participants had children and worked at the time of the interview study. Further characteristics of the interview participants can be seen in Table 2. Since HCP patients and their relatives are a relatively small group in Germany, characteristics such as the role of the interview participant, their gender and age are not indicated in the following quotes to guarantee anonymity. More information about the study results is available from the first author upon request.

**Table 2.** Sample characteristics.

Characteristics	Patients (*n* = 17)	Relatives (*n* = 7)	All (*n* = 24)
Age	20–70 (median: 49)	47–78 (median: 67)	20–78 (median: 52.5)
Age groups			
18–30	2		2
30–50	7	1	8
50–70	7	5	12
70–90	1	1	2
Gender			
Male	7	3	10
Female	10	4	14
Genetically tested	11		11
In acute episode	1		1
Education			
A-level	10	2	12
Secondary school	5	2	7
Other	2	3	5
Marital status			
Single	5		5
Married	11	7	18
Living together	1		1
Has children	12	7	19
Employment	13	5	18
Member of patient organization	11	3	14
Relationship to patient			
Parent		3	3
Spouse		4	4

Reprinted: Müller et al.^64^.

Four topics were chosen as the focus of the current paper due to the richness of the results: (1) The unpredictable clinical course of HCP; (2) HCP as a devastating experience; (3) HCP as part of a normal life; and (4) being reduced to HCP.

### The unpredictable clinical course of HCP

The study revealed that those affected by HCP experienced the illness as an ongoing but unstable and unpredictable condition. The participants described that the acute phases of the illness always return, likening it to a cycle. They emphasized, additionally, that the course of the illness could not be predicted. The participants could not say when and how long the acute phases would last. Phases of one to several days were reported. Some participants experienced several phases in short intervals, others no acute phases for many years. The participants reported uncertainty and feelings of powerlessness regarding the acute phases because they could not say what caused an impending exacerbation. In addition, from their perspective, nothing could be done in advance against becoming symptomatic again. Since they could influence neither the occurrence nor the course of the acute phases, both patients and family members felt helpless and at the mercy of the illness.

You just got over it, and then it started again. [Interview 15]

We live on a powder keg. We don’t know when it will come because it is so, well, unpredictable. It can go bad; it can go well for a long time. [Interview 17]

Some participants said that they were always vigilant of new episodes. They highlighted that they always had to be prepared for potential acute phases. One participant reported, for example, that the laundry was constantly done so that everything was ready should an acute phase of the illness come. Relatives particularly referred to an increased attention and alertness in their daily lives. One relative, for example, reported phases in his/her family life, in which he/she continuously paid attention to the noises at night to hear if there might be something wrong with the family member affected, even if he/she was not in an acute phase of the illness.

Well, a certain fear is stored somewhere inside yourself that now, suddenly, a phase will come, and you would be at the mercy of it again. Yes, you’re always a little bit on guard. [Interview 11]

The participants also indicated various restrictions and turning points in their lives due to the unpredictable character of the illness, for example, in terms of education, job fulfilment or family planning. Other aspects of life in which the participants felt restricted by the unstable course of the illness extended to vacation plans, going abroad, sports, leisure and social activities. The participants reported that they had had to cancel their plans or appointments due to acute phases and that it was difficult to plan anything at all.

At the beginning, I dare not go anywhere. Now, I can’t go on holiday with my grandchildren alone because if I had such a phase somewhere […] it would be a shock for them [the grandchildren]. [Interview 3]

At university, I had been promised that I could go to the USA, but due to the illness, which occurred for the second or third time, there were problems with the health insurance […] that was also a limitation, which hurt me very much. [Interview 4]

### HCP as a devastating experience

The acute phases were described very differently by the participants, ranging from mild to very severe. The severe phases were usually described as lasting a few days, but one participant also spoke of several weeks. Again, the participants could not say with certainty what had triggered an acute exacerbation. In the case of the latter, the participants reported that they were extremely weak. They described, for example, a rapid loss of physical energy and feelings of being ineffective and impassive. Furthermore, they could no longer eat and drink and, in the worst case, had had to go to the hospital. The description often focused on extreme pain, which could not be treated but was actually unbearable. The pain and weakness particularly brought them to their physical and psychical limits.

The participants who had experienced a severe phase designated it as a disruptive experience. They described it as devastating, very frightening and reported fear of death as an example. Furthermore, they emphasized that the severe phases took them out of their everyday life, for example, from work, that they had no longer been able to do anything and that the severe phases are very difficult to endure.

This [the acute phase] is really a point where you think, well, it can’t go on. […] and you can’t really go back into life because you always have some pain and so on and you don’t know what's going on now. That worries you. [Interview15]

Family members expressed similar feelings regarding severe phases. When acute phases occurred, relatives were very concerned about the patient's well-being and afraid that the phases could worsen. Some reported concern about repeated visits to the hospital and physicians; others stated the fear of the patient's death. Relatives who had observed the patient's suffering reported that the severe phases would be extremely difficult to bear for them.

### HCP as part of a normal life

The participants also experienced long episodes in which the illness remained unremarkable and unnoticed. Some participants reported no acute phases for several years or even decades. The participants emphasized that the illness disappeared after acute phases and explained that their lives were then comparable to those of healthy people. Several participants did not label themselves or their relatives as being ill but, on the contrary, as being healthy. Parents particularly did not want to talk about their children as being ill.

But as soon as I’m out of the hospital and go back into everyday life and realize, ah, everything is fine and everything is the same as with everyone else, then it's hard for me to say, yes, I have an illness, because it's not present at that moment. [Interview 5]

In addition, the participants regarded HCP as an inevitable part of their existence, as a part that has always been part of their lives because nothing could be done about it. Some participants saw HCP as an essential component, which had made them the person they are today. In several interviews, the participants relativized restrictions and difficulties, which they had mentioned previously. Comparisons to other conditions, such as cancer, were often used to relativize HCP and the associated burdens.

On the other hand, our neighbor has pancreatic cancer now. By comparison, I’m fine at my age. Or when I was in rehab and saw the problems of others, I told myself, I have nothing bad at all. [Interview 3]

### Being reduced to HCP

Some participants criticized that others tended to reduce those affected to their illness and the associated aspects. They experienced that other people only noticed the disease and not the person or the current context of the person's state of health and illness. One participant reported, for example, that once he/she had mentioned the disease, the conversation partner only wanted to talk about HCP, although the participant him/herself would have preferred to talk about other topics. Another example was the participants’ experience in healthcare, particularly during medical examinations. They reported that other health issues had been overlooked by the medical staff as they focused exclusively on the pre-diagnosed HCP.

[…] and you’re often reduced to the disease […] this is often worse for me than anything else. So, this is sometimes forgotten a bit, that you can be a normal person in addition to the disease and still have other problems […]. So, if I just go to a doctor now and say I have the disease, then he just looks at me at this point and at nothing else. I always say, yes, but I also have other things. That is, I think, very, very important. [Interview 5]

In this context, the participants spoke about expectations regarding the patients’ behaviour, which often came with the attribution of illness. Some participants had experienced, for example, that others expected them to eat healthily, not to drink alcohol, smoke or do risky sports. One participant, for instance, stated that in his/her childhood he/she had been excluded from sport because of HCP, even though he/she would have been able to attend sports classes.

## Discussion

The results present four categories describing the subjective experience of those living with HCP and show particularly the unpredictable dimension of living with the illness. The findings show that HCP is an illness with a very unstable character whose manifestation can range from mild to very harmful experiences. Although their interview study focuses on acute pancreatitis, the results of Boije et al.^23^ confirm the wide variation of the intensity and duration of acute pancreatic phases. Furthermore, the participants described feelings of uncertainty, anxiety and fear due to the lack of knowledge regarding why and at what time the pancreatic attack had occurred.^23^ In a previous survey by Shelton et al.^24^ participants with hereditary pancreatitis (HP) expressed similar feelings, describing the worry and uncertainty about when an acute phase will occur. Moreover, feelings of helplessness were described by both the patients regarding their own disease and relatives observing the patients’ suffering.^24^ The participants in the present study confirmed these findings by reporting fear, uncertainty and helplessness due to the unplannable and sudden experiences of the acute phases.

The impact on health-related quality of life, for example, regarding daily activities and psychosocial well-being, described in the survey by Shelton et al.^24^ were echoed in the current study, demonstrating restrictions regarding social activities, education and job fulfilment. Related findings have been described in the interview study by Boje et al.^23^ indicating that the physical suffering of pancreatic attacks has adverse effects on every day and social life. A recent qualitative study with CP patients by Cronin and Begley^22^ highlights the permanent experience of disruption at the physiological, social and psychological level. By contrast, participants in the current study depicted phases of exacerbation but, in between, the disease was predominantly invisible.

In the current study, both patients and family members have described the acute severe phases as a devastating experience. This disturbing dimension of the illness can be found in other studies. Although in the context of genetic testing of HP, both a survey by Applebaum-Shapiro et al.^26^ and the one by Shelton et al.^24^ refer, for example, to the ‘disturbing nature of seeing relatives affected with HP.’ At first glance, the description of the devastating experience by the participants in the present study is reminiscent of Bury's concept of biographical disruption.^45^ According to Bury, the onset of a chronic illness separates the patient's life into a lifespan before and after illness. In the study with CP patients by Cronin and Begley, the participants described such a shift from a well person to a person with CP.^22^ The unplanned and sudden transformation from being healthy to being in an acute phase were also described in the study with patients with acute pancreatitis by Boije et al.^23^

However, the participants in the current study did not report such a clear transition. They spoke instead of recurring disruptive moments as part of their ongoing biography. The disruptive dimension of HCP refers neither to the participants’ entire biographies, nor to a single point in their lives, but rather to the recurring difficulty of integrating the acute illness phases into daily life. The concept of biographical disruption by Bury, thus, cannot completely mirror the viewpoints of individuals affected by HCP. These findings are in accordance with several studies which show that the concept of biographical disruption is only relevant to the experience of chronic illness to some extent.^46–49,54^

Most participants in the current study had grown up with the diagnosis of HCP and/or were already familiar with the illness because of its occurrence in the family. However, even if familiar with or expected, the acute phases could be disruptive. The unpredictability of the phases was, besides their strength, an important reason for this. Patients with acute pancreatitis similarly described the burden of the unplanned and sudden occurrence of the acute phases, which includes shocking and unreal sensations.^23^ The experience of HCP patients is, thus, in accordance with the concept of biographical contingency.^49^ This concept describes life with a chronic illness as normal, which means undisturbed, to a large extent. Since the chronic illness is only experienced from time to time, the biographies and the daily routines are disrupted only momentarily.^49^ By describing life with a chronic illness as normal and, at the same time, granting the disease a disruptive potential, the concept of biographical contingency covers the dimensions expressed by the study participants adequately.^49^

Altogether, the study reveals that HCP can be understood neither as a linear predictable path nor as a dichotomy of life before and after illness but as a continuous, constantly shifting process. This description is covered by Paterson's ‘shifting perspectives model’ of chronic illness.^50^ As described in the current interview study, the perspectives of the participants can shift in the model from illness (i.e. an acute phase is in the foreground) to wellness (i.e. HCP is largely unnoticed) and *vice versa*.^50^ Paterson's model helps to resolve the seemingly contradictory statements of the participants. Several participants, for example, stated that living with HCP was never normal because they always had to be vigilant about acute phases. At the same time, the participants said that the disease had disappeared after the acute phases and then they led a normal life. In addition, the illness in itself and the associated difficulties were often relativized throughout the interviews. Paterson's model can cover these variations in the participants’ attention to HCP and meets the individual character of the illness experience.

The ethical problem of being reduced to HCP is linked with the shifting process. The changing character of HCP can lead to diverging perceptions. Because the illness is not always present, participants describe themselves as healthy, whereas others label them as ill. This misattribution can be seen as a form of pathologization.^55–57^ The experience of being reduced to the illness and labelled as ill is described by the study participants as problematic because the attribution often leads to expectations regarding the participants’ behaviour and can even pave the way for a depersonalization or objectification of the participants. A reductive view can lead to severe problems for the individual in the healthcare system, for example, when other diseases or symptoms are overlooked. In addition, conflicts can arise if the perceptions of those affected and healthcare professionals diverge and patients or their relatives do not behave as expected by the healthcare professionals.^58^ The experience of being reduced to the illness could be prevented in the context of the healthcare system by focusing on the patient and his/her interests rather than the disease. The exchange with other affected patients and family members could provide further assistance, especially in dealing with feelings of helplessness, being at the mercy of the illness and reduced to it. Consequently, a next step could be to develop a program of psychological support for HCP patients and their families and to provide more support for different forms of patient self-help.

A further step to develop better care and support for those living with HCP could be to ensure long and constant but, at the same time, phase-specific support. Trustful collaborations between patients, families and healthcare professionals are essential for high-quality care, especially in the context of long-lasting chronic conditions.^58,59^ A better understanding of the shifting character of HCP and the associated problems can help healthcare professionals to establish a trustful relationship and provide sustainable support. In addition to trustful and permanent support, specific assistance in the respective phases is very important. Consequently, it should be ensured that the knowledge of the changing character of HCP is integrated into the scientific and practical education of healthcare professionals.

## Strengths and limitations

The current study was designed to elicit a deeper understanding of living with HCP and, as far as the authors are aware, it is the only study of this kind. One strength of this study is the use of semi-structured interviews because they allowed more in-depth information and provided detailed insights into how those affected experience HCP. Another strength is the inclusion of both patients and their relatives. Partners and family members often added further information to the findings. Maximum variation sampling was used to ensure the inclusion of participants of differing gender, in different parts of their lifespans and with varying levels of HCP. HCP is a rare disease. The prevalence of the disease and the difficulty in diagnosing and recruiting HCP patients and their families for a research study, therefore, limits the sample size of this study. The participants were contacted via a patient organization, thus, it is possible that the participants were reluctant to make comments that might be perceived as critical about the support of the organization. The recruitment via the patient organization also resulted in a slight majority of patient organization members among the individuals interviewed. Individuals with HCP who were not members of the organization were much more difficult to contact by the research team and, therefore, represent a smaller proportion in the sample. The membership of an organization could indicate a more ‘engaged’ cohort.

It was not possible for two participants to conduct the interviews at home. These interviews were, therefore, conducted by telephone. There are differences in the data collection between face-to-face interviews and interviews by telephone and an important and unresolved issue about social desirability bias generated through telephone interviews.^60^ The nuances of body language, for example, and other nonverbal cues associated with face-to-face interaction may be lost over the telephone, and trust is difficult to establish.^60^

Furthermore, the participants’ medical conditions might have had an influence on the study results. Only one of the participants interviewed was in an acute episode at the time of data collection. Talking from a place ‘outside their disease,’ the participants might have reported other aspects than they would have had in an acute phase. Finally, the study does not have a longitudinal design but instead reproduces the participants’ views at a particular point in their lifespan. Longitudinal qualitative research with repeated interviews throughout could provide further information on the subjective experience of HCP. The analysis of qualitative data is not a straightforward process, often accompanied by concerns, e.g. on reliability and generalizability, and there are different opinions about which criteria are the best for evaluating the trustworthiness of qualitative content analysis.^61–63^ Concerns related to trustworthiness are minimized in the current study by several strategies, such as protocolling the different stages of the analysis, regular reflective discussions within the research team and full reporting of the process of data analysis. In addition, researchers with different disciplinary backgrounds were part of the study team to mitigate assumptions and bias during data analysis.

## Conclusion

The current paper presents findings on the subjective experience of patients with HCP and their relatives showing implications resulting from HCP as a chronic but constantly changing condition. A better understanding of the unpredictable and shifting character can help healthcare professionals to tailor the care to meet the needs of those affected. Individual support for HCP patients should be patient-focused, cover psychological support and be carried by both the healthcare system and the social network, for example, patient self-help groups. Further research should investigate what specific forms of support HCP patients and their families need and how the different forms of support can help in the acute phases, affect the phases between the acute attacks, and help to deal with the problem of pathologization. The focus of the current study is on the experiences of HCP, but the issues discussed are potentially relevant to other chronic conditions that are variable in their nature. Further research should address how the unpredictable and constantly changing character of chronic conditions can be better considered in the research and development of therapies and the scientific and practical training of healthcare professionals.

## Supplemental Material

sj-pdf-1-chi-10.1177_17423953211039774 - Supplemental material for Lived Experience of Hereditary Chronic Pancreatitis – A Qualitative Interview Study Click here for additional data file. Supplemental material, sj-pdf-1-chi-10.1177_17423953211039774 for Lived Experience of Hereditary Chronic Pancreatitis – A Qualitative Interview Study by Regina Müller, Ali A Aghdassi, Judith Kruse, Markus M Lerch, Christoph Rach, Peter Simon and Sabine Salloch in Chronic Illness
